# Predictors of Mortality in *Pseudomonas aeruginosa* Bloodstream Infections: A Scoping Review

**DOI:** 10.3390/pathogens15010061

**Published:** 2026-01-07

**Authors:** Kartini Abdul Jabar, Nur Izzatul Auni Romli, Kumutha Malar Vellasamy, Vinod Pallath, Anis Rageh Al-Maleki

**Affiliations:** 1 Department of Medical Microbiology, Faculty of Medicine, Universiti Malaya, Lembah Pantai, Kuala Lumpur 50603, Malaysiakumuthamalar@um.edu.my (K.M.V.);; 2 Jeffrey Cheah School of Medicine and Health Sciences, Monash University Malaysia, Bandar Sunway 47500, Selangor, Malaysia

**Keywords:** *Pseudomonas aeruginosa*, bloodstream infections, risk factors, mortality, post-COVID-19 pandemic

## Abstract

*Pseudomonas aeruginosa* bloodstream infections (PABSIs) are a major clinical challenge due to their association with significant mortality and antimicrobial resistance mechanisms. The COVID-19 pandemic changed antimicrobial practices, intensive care management, and patient risk profiles, potentially influencing the epidemiology and outcomes of PABSIs. In the post-pandemic period, practices were expected to revert to normal. The objective of this scoping review was to identify and summarize reported mortality rates and risk factors for PABSIs in studies published between 2023 and 2025. Literature searches were conducted across PubMed, Web of Science, Embase, and Scopus. Screening was performed in accordance with PRISMA-ScR guidelines. Twenty-two eligible studies were included. Mortality rates varied across the study setting and populations; however, several consistent predictors were consistently identified, including carbapenem exposure, multidrug-resistant *Pseudomonas aeruginosa*, hematologic disease or malignancy, corticosteroid therapy, sepsis or septic shock, mechanical ventilation, and higher severity-of-illness scores. Few studies have linked molecular mechanisms to patient outcomes, highlighting important gaps in knowledge. Notably, only a small number of studies included the post-pandemic period but did not analyze the data separately. Despite limited available evidence, critically ill and immunocompromised patients remain at greatest risk of death from PABSIs. This review highlights the need for a broader comparative analysis in future.

## 1. Introduction

*Pseudomonas aeruginosa* (*P. aeruginosa*) is an opportunistic pathogen associated with significant morbidity and mortality, particularly in hospitalized and immunocompromised patients [1]. Its ability to thrive in moist healthcare environments, form biofilms, evade host immunity, and exhibit intrinsic and acquired antimicrobial resistance makes it one of the most challenging Gram-negative organisms to manage clinically [1,2]. Bloodstream infections caused by *P. aeruginosa* (PABSIs) lead to morbidity and mortality, with mortality rates for PABSIs ranging from 18% to 53%. PABSIs have been associated with increased mortality relative to bloodstream infections caused by other Gram-negative pathogens or *Staphylococcus aureus* [1,3].

The COVID-19 pandemic caused profound disruptions to global healthcare systems that have reshaped the epidemiology and management of bacterial infections. During the pandemic, unprecedented pressures on intensive care units, extended mechanical ventilation, widespread use of corticosteroids and immunomodulatory therapies, and the extensive empirical use of broad-spectrum antibiotics created an environment conducive to nosocomial infections, including those caused by *P. aeruginosa* [4,5,6]. These changes have had downstream effects on antimicrobial resistance patterns, patient vulnerability, and healthcare-associated infection risks [7]. The World Health Organization (WHO) declared the end of the pandemic in May 2023; the post-pandemic period thus presents a critical window to re-evaluate PABSIs in light of these shifts, in addition to an opportunity to determine whether pandemic-related trends have persisted, stabilized, or reversed [8].

Despite the clinical importance of PABSIs, there remains a scarcity of consolidated post-pandemic evidence describing recent predictors of mortality and acquisition. As healthcare systems transition into the recovery phase in the post-COVID-19 pandemic era, updated insights are urgently needed to inform antimicrobial stewardship, infection prevention strategies, diagnostic pathways, and resource allocation.

Although studies conducted before and during the COVID-19 pandemic identified established mortality predictors such as severity of illness, antimicrobial resistance, and antibiotic management [9,10,11], it remains unclear whether these risk factors have changed in magnitude or relevance in the post-pandemic setting. This scoping review was therefore conducted to map the current (2023–2025) evidence on PABSIs in the post-COVID-19 era. Specifically, we aim to (1) identify recent predictors of mortality in PABSIs, (2) describe risk factors associated with acquiring PABSIs, (3) summarize recent mortality rates across diverse clinical settings, and (4) characterize the geographical distribution of published research. This review provides a foundation for future comparative studies across pre-, during-, and post-pandemic periods and highlights knowledge gaps requiring further investigation. Ultimately, these findings serve to support clinicians and researchers in navigating the evolving landscape of PABSIs and improving patient outcomes in a rapidly changing healthcare environment.

### Rationale for Conducting the Study in the Post-Pandemic Period

The COVID-19 pandemic profoundly reshaped healthcare systems worldwide, altering patterns of antimicrobial use, infection control practices, ICU resource allocation, and patient vulnerability profiles [4,7]. These unprecedented changes have had substantial downstream effects on the epidemiology of bacterial infections, including PABSIs. In previous studies, researchers have identified an upsurge in *P. aeruginosa* infections during the pandemic [12,13]. We postulate that the post-pandemic era would be marked by a decline and thus a change in prescribing practices and prevalence. Although a significant number of studies were published during the pandemic, very few authors distinguished pre-pandemic, pandemic, and post-pandemic data. By focusing specifically on the post-COVID-19 period (2023–2025), this scoping review fills this critical knowledge gap and lays the groundwork for future comparative analyses across the three periods: pre-pandemic, pandemic, and post-pandemic.

## 2. Methods

### 2.1. Search Strategy and Data Sources

The Preferred Reporting Items for Systematic Reviews and Meta-Analyses Extension for Scoping Reviews (PRISMA-ScR) guidelines were followed in the development of the protocol for this review. However, this review was not registered, in accordance with common scoping review methodology and consistent with PRISMA-ScR guidelines. A completed PRISMA-ScR checklist has been provided in the Supplementary Materials (Supplementary Table S1).

This review was performed to assess the current literature published in the post-COVID-19 pandemic period to identify the gaps in current knowledge and as a precursor to a systematic review to compare the findings between the three periods, namely, the pre-pandemic, pandemic, and post-pandemic eras.

Comprehensive searches were conducted across four databases: PubMed, Web of Science, Embase, and Scopus. The keywords and MeshTerms used have been provided in the Supplementary Materials (Supplementary Table S2). The keywords (“*Pseudomonas aeruginosa*” OR (*P. aeruginosa*) AND (“Bloodstream Infections”) OR (“Bloodstream Infection”) OR (“Bacteremia”) AND (“Mortality”) were used to search for articles. Only articles published between 2023 and 2025 were included in this review. Searches were performed on 27 November 2025 and 8 December 2025.

### 2.2. Study Selection

Publications were considered eligible if they met the following criteria: (1) published between 2023 and 2025, (2) original research articles involving adults, (3) contained clinical data relevant to mortality predictors of PABSIs, (4) written in English, and (5) full-text available. However, we did not include articles that were non-original research papers or without peer-review, such as letters, reports, meta-analyses, duplicate studies, perspectives, commentaries, opinions, and conference proceedings, those not in English, and those without full-text or extractable data. Screening of titles, abstracts, and publication years was performed according to predefined criteria, with publications before 2023 excluded at this stage. Following this process, 427 articles were removed during title and abstract evaluation, leaving 43 studies for full-text review. These full texts were independently reviewed by two authors, with any disagreements resolved by consensus or the involvement of a third author.

### 2.3. Data Extraction and Synthesis

Data were charted independently by two reviewers using a structured Excel extraction sheet. The simple standardized Excel sheet was developed before the review. Extracted variables included study site, period of study, clinical setting, population size, predictors of mortality, predictors of acquiring PABSIs, and mortality rates. For studies that were reviewed at the title and abstract levels, the keywords “*Pseudomonas aeruginosa*” and “bloodstream infections” or “bacteremia” were used for screening. All titles, abstracts, and full texts were reviewed by two authors. Any discrepancies between reviewers were resolved through discussion or the involvement of a third author. As the findings of the studies were heterogeneous, a narrative synthesis was generated.

## 3. Results

### 3.1. Characteristics of Included Studies

**Figure 1:**
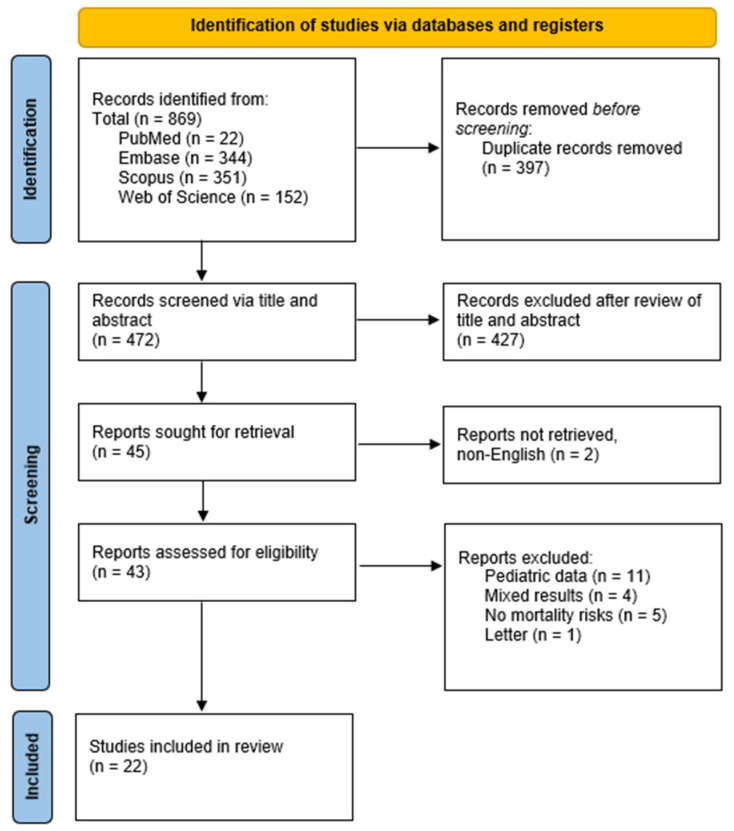
PRISMA flow diagram.

A total of 22 articles were identified from PubMed, 152 from Web of Science, 344 from Embase, and 351 from Scopus. After removing 397 duplicates, 472 records remained for screening. Titles and abstracts were independently screened based on the review of keywords, leading to the exclusion of 427 articles. The remaining 43 articles were assessed for eligibility after their full texts were obtained. After screening the remaining 43 articles, only 22 studies were included in the review that fulfilled the inclusion criteria. Details of the studies have been included in Table 1. The synthesis of the review is provided in Figure 1.

A total of 14,648 pieces of patient data or 18,652 PABSI episodes were reviewed and analyzed in this scoping review. All 22 cohort studies used retrospective, observational, cohort designs. Most studies were conducted in single-center setting (14/22) and eight studies were multicenter or multinational.

### 3.2. Geographic Distribution of Studies

**Figure 2:**
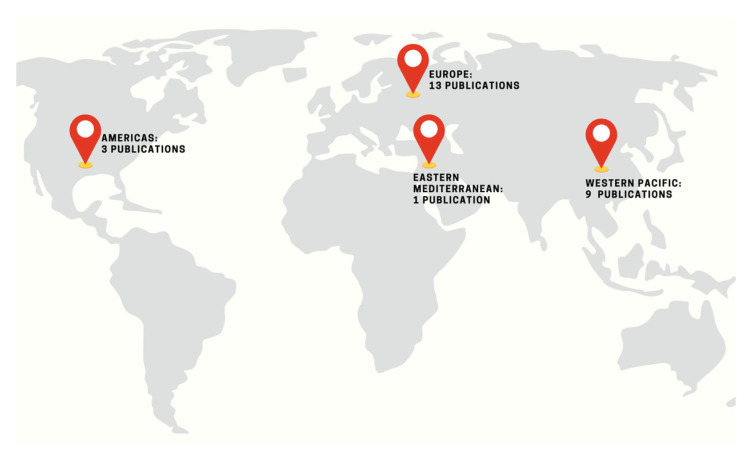
World map of publications by region.

The map (Figure 2 and Table 2) illustrates that the majority of the studies originated from Europe (13 publications, 59%) [14,15,18,19,22,23,24,26,27,30,33,34,35] followed by the Western Pacific Region with nine publications (30.9%) [14,16,17,21,24,25,29,31,32]. These findings indicate strong research activity within these regions. The Americas were responsible for three studies (13.6%) [20,23,28] and the Eastern Mediterranean Region was involved in one study (4.5%) [23]. Overall, the map highlights that research is globally distributed, with under-represented areas including Africa and the Southeast Asia Region.

Several countries were major contributors to the global distribution of publications, such as Spain (5 studies, 22.7%) [14,15,23,27,34], China (5 studies, 22.7%) [17,25,29,31,32], Italy (4 studies, 18.2%) [22,23,26,30], and Türkiye (3 studies, 13.6%) [18,19,23]. Several studies [14,23] involved multinational centers with data collected across the Americas, Europe, and Eastern Mediterranean Regions.

Overall, the global distribution of publications demonstrates that the research output on this topic is globally sourced but uneven across the WHO regions, with certain countries and regions with higher research output than others.

### 3.3. Predictors of Mortality in PABSIs (2023–2025)

The literature published between 2023 and 2025 revealed a broad range of predictors associated with mortality in patients with PABSIs, as highlighted in Table 3. These predictors are clustered into several major domains: severity-of-illness indicators, microbiological or antimicrobial-related factors, host-related conditions and biomarkers, infection source-related characteristics, and other contextual clinical factors.

#### 3.3.1. Severity-of-Illness Predictors

Markers of acute physiological deterioration were the most frequently identified predictors of mortality. Sepsis, septic shock, or shock at presentation were reported in seven studies [15,22,25,26,29,30,35], making them the most consistently observed predictors across the literature. Mechanical ventilation, a reflection of severe respiratory compromise, was noted in five studies [18,23,31,32,34], and the use of extracorporeal membrane oxygenation (ECMO) was reported once [18]. Several well-established severity scoring systems were also strongly associated with mortality: APACHE II (three studies) [20,21,28], Pitt bacteremia or qPitt score (three studies) [17,18,22], and SOFA score (one study). Additional severe illness indicators, such as multiple organ failure (two studies) [17,29] and the need for inotropic support (one study) [18], further highlighted the role of critical physiological instability in determining poor outcomes.

#### 3.3.2. Microbiological and Antimicrobial Predictors

Three studies [15,16,25] reported MDR or CRPA as significant predictors of mortality, highlighting the major impact of antimicrobial resistance on clinical outcomes. Prior carbapenem exposure, identified in three studies [23,31,34], also increased mortality risk, likely due to selection pressure favoring resistant strains. Less frequently reported but important antimicrobial-related predictors included prior colistin use [29], variations in cefepime dosing [28], probability of target attainment (PTA) > 65% [21], and inappropriate or inadequate empirical antibiotic therapy [23,34], each reported in a single study. In one study, high-risk genotypes (ST175 and ST235) were identified as poorly prognostic, suggesting a role for bacterial lineage in the severity of infection [24].

#### 3.3.3. Host-Related Conditions and Biomarkers

Host vulnerability played a major role in determining mortality risk. Older age, identified in four studies [14,15,25,30], remained a consistent predictor. A high comorbidity burden, assessed using the Charlson Comorbidity Index, appeared in three studies [19,20,35]. Similarly, hematologic malignancies or hematological disease [17,25,34] and corticosteroid therapy [14,25,32] were each identified in three studies, reflecting increased susceptibility in patients with compromised immune function.

Inflammatory and hematologic biomarkers also contributed to risk. In two studies, elevated inflammatory markers (PCT, CRP, and D-dimer) [18,25] and thrombocytopenia [18,19] were identified in each as predictors of mortality. Other single-study predictors included dementia [18], acute kidney injury [19], G-CSF use [23], low hemoglobin [32], and coronary artery disease [31], reflecting the broad systemic impacts of chronic or acute medical conditions in worsening prognosis.

#### 3.3.4. Infection Source-Related Predictors

In three studies, the authors reported that hospital-acquired or hospital-onset infections were associated with higher mortality, likely due to exposure to resistant strains and delayed recognition [14,20,30]. High-risk infection sources, such as pneumonia or severe abdominal infection [15,30], and urinary tract infection [23,34] were noted in two studies each and were strongly linked to poor outcomes. Specific infection origins, such as internal organ infection, namely, pneumonia or complicated intra-abdominal infection [33], lower respiratory tract infection [35], primary bloodstream infection [19], and persistent bloodstream infection [23] were each reported in single studies. In addition, urinary catheter use [15] was reported as a risk factor in a single study, reinforcing the importance of device-associated infection prevention.

#### 3.3.5. Other Clinical Predictors

A small number of additional factors were reported, demonstrating the complexity of PABSI outcomes. Lack of adequate source control was associated with mortality in two studies [34,35], with delayed catheter removal (>48 h) [34], infections occurring during the COVID-19 pandemic [19], and facility complexity level [20] each reported once.

### 3.4. Predictors of Acquiring PABSIs

A wide range of clinical, microbiological, and healthcare-related predictors associated with the acquisition of PABSIs were reported with varying frequency across the literature, reflecting different study populations and clinical contexts. Overall, the most consistent predictors were related to antimicrobial exposure, immunosuppression, hematologic conditions, catheter-related factors, and indicators of severe systemic illness, as shown in Table 4.

The most frequently reported predictor across all studies was carbapenem-related exposure, including prior carbapenem therapy or exposure within 90 days. This risk factor was reported in five studies [15,17,18,25,29], highlighting the strong association between carbapenem use and subsequent development of PABSIs.

Severe neutropenia (ANC < 100/mm^3^) was the second most common predictor, identified in two studies [25,34], highlighting immunosuppression as a major vulnerability. Additional predictors observed in single studies further emphasized immunocompromised states as high-risk conditions, including a history of allogeneic hematopoietic stem cell transplantation (allo-HSCT) [25], hematologic malignancy [15], immunosuppressive therapy [17], and older age [25]. Receipt of antifungal prophylaxis was also noted once [25], likely reflecting the broader immunosuppression in this patient population rather than a direct causal effect.

Catheter and device-related factors formed another important group of predictors. Catheter duration longer than seven days [34], phlebitis [34], and central venous catheterization [31] were individually associated with increased risk. Several microbiological indicators identified are positive cultures in both vials, short time-to-positivity (TTP < 13 h), and differential TTP < 2 h [34]. These findings reinforce the significant role of intravascular devices as a source and route of PABSI acquisition and the importance of microbiological indicators.

Markers of clinical severity were also identified as predictors. Septic shock at BSI onset [34], multiple organ failure [29], and elevated inflammatory markers such as C-reactive protein [18] appeared in single studies. Although less common, these indicators suggest that patients experiencing systemic deterioration are more susceptible to bloodstream invasion, particularly in the context of pre-existing infection or colonization.

In several studies, prior healthcare exposures were identified as contributing factors. Prior ICU hospitalization was highlighted once [17], suggesting that high-intensity care environments increase risk through device use, antimicrobial pressure, and exposure to resistant organisms. In addition, prior colonization or infection with CRPA was identified as a predictor [29], reflecting the importance of colonization pressure in subsequent bloodstream invasion.

Other, less frequently documented predictors included chronic lung disease [29], transplantation [29], and active antibiotics at the onset of bacteremia [34], each reported in one study. These findings illustrate the diversity of clinical conditions and exposures associated with PABSI acquisition, although their individual significance may vary depending on the patient population and healthcare setting.

### 3.5. Mortality Rates

Across all included studies, a total of 14,648 individual patients or 18,652 bloodstream infection episodes were reported, reflecting substantial variation in study size, design, and reporting methods.

Mortality endpoints varied across studies and included 7-day, 14-day, 28-day, and 30-day all-cause mortality, depending on the individual study design. Mortality rates varied considerably across the studies, influenced by differences in patient demographics, clinical severity, resistance patterns, and healthcare settings. Almost all studies provided 30-day mortality rates [14,15,16,17,18,19,20,21,22,23,24,26,27,28,29,30,31,32,33,34,35], with some including 7-day mortality [23,24,27,31] or 14-day mortality rates [35] and one study reporting 28-day mortality [25]. Atamna et al. (2023) [14] included 464 elderly patients (≥80 years) and reported a 30% mortality rate, highlighting increased vulnerability in older populations. Sakaguchi et al. (2023) [16] reported an all-cause mortality of 32.9% among 48 patients, reflecting high mortality even in smaller cohorts. Yuan et al. (2023) [17] documented 274 patients and noted mortality differences by resistance type. The mortality rates were 39% in CRPA and 50% in difficult-to-treat resistant strains (DTRPA), thus indicating the strong impact of antimicrobial resistance on survival.

Across studies, the variation in reporting timeframes, outcome measures, and cohort sizes indicates the heterogeneity of available evidence. Study sample sizes ranged widely, from small cohorts of 40–80 patients [16,21,22,31,33] to multicenter datasets exceeding 1000 patients [20,23,34]. This diversity influences the interpretability of pooled mortality trends but consistently demonstrates that mortality associated with PABSIs remains high across different settings, frequently surpassing 20–30%.

Overall, a consistent pattern can be seen across studies. PABSI is associated with substantial mortality, especially in older adults, critically ill patients, or infections caused by resistant strains. The marked differences in patient numbers and outcome measures across studies highlight the need for standardized reporting and larger, multicenter investigations to better understand mortality risks and improve clinical outcomes.

## 4. Discussion

The findings of this scoping review highlight the predictors of mortality and acquiring PABSIs. Carbapenem exposure emerged as a central risk factor influencing both acquisition and mortality, suggesting that antibiotic prescribing practices continue to shape the epidemiology of these infections. Although the studies reviewed were investigated in different clinical contexts, such as the study by Royo-Cebrecos (2024) [23], which was conducted among hospitalized patients, including those with solid tumors and hematologic malignancies, that of Guo et al. (2025) [31] in hematology and ICU populations, and that of Marco et al. (2025) [34], specifically in catheter-related bacteremia, they consistently identify carbapenem exposure as a significant mortality predictor. This consistent association indicates that antibiotic prescribing practices continue to shape the epidemiology and severity of PABSIs across different clinical settings. These findings are further supported by pre-pandemic evidence [36,37]. Buehrle et al. (2017) [36] reported that 57% of patients received carbapenems prior to developing *P. aeruginosa* bacteremia, although carbapenem exposure was not identified as a mortality risk in that specific cohort. In contrast, the authors of another pre-pandemic investigation identified carbapenem exposure as the only major risk factor for PABSIs and found a distinct survival difference, with none of the survivors noted as having prior carbapenem exposure, whereas half of the non-survivors had [37]. This relationship highlights the need for robust antimicrobial stewardship programs, especially in high-risk units.

The presence of CRPA was strongly linked with increased mortality. Yuan et al., 2024 [25], specifically studied CRPA BSI and observed a more than 3-fold difference in 28-day mortality between the CRPA BSI group and the CSPA BSI group (38.2% and 12.2%), respectively. This risk factor aligns with the results of many studies conducted before the pandemic, in which two different meta-analysis studies performed on mortality attributable to CRPA bacteremia highlighted the negative impact of CRPA on mortality in PABSI [38,39]. Other pre-pandemic studies that are not included in the meta-analysis also provide evidence supporting this finding [40,41]. Limited therapeutic options for CRPA, coupled with frequent delays in effective treatment, likely contribute to this outcome [42]. Moreover, resistance may be associated with enhanced virulence in certain strains, although the authors of the studies included in this review did not investigate molecular–clinical correlations in depth.

Critically ill patients, including those with high APACHE II [20,21,28], Pitt [17,18,22] or SOFA scores [14], and sepsis [15,22,25,26,29,30,35], consistently demonstrated increased risk of mortality. As APACHE was established as the gold standard for severity of disease classification system [43], in multiple studies dating back to two decades before the COVID-19 pandemic, the authors consistently found high APACHE scores to be a crucial predictor of PABSI mortality [44,45]. The Pitt bacteremia score [46], a scoring system established specifically for bacteremia, is also a popular scoring system used to predict mortality combined with APACHE. As such, in many studies conducted in the pre-pandemic period, the authors also reported the Pitt score to be an important risk factor for mortality [36,37]. SOFA score was subsequently developed for sepsis; therefore, it is not frequently included in bloodstream infection studies in comparison with the APACHE and Pitt scoring systems [47], which aligns with only one study in our review, in which SOFA was found to be a mortality predictor for PABSIs. Alternatively, some studies include SOFA score to predict PABSI mortality beyond 30-day mortality, such as 90-day mortality, although not necessarily a significant predictor [48,49]. These findings accentuate the importance of early recognition, aggressive management, and timely antimicrobial therapy in improving outcomes for high-risk individuals [1].

All mortality predictors discussed in this scoping review were based on multivariate analysis by the individual studies, except for urinary tract infection by Marco et al. (2025) [34] and elevated inflammatory markers (PCT/CRP/D-dimers) by Çaydaşi et al. (2024) [18] which were univariate analysis.

Mortality rate variations across the studies are driven by a combination of patient- and pathogen-related factors. Older age, immunosuppression, and co-morbidities such as hematologic disease or malignancy, would consistently increase vulnerability to severe sepsis and poor outcomes [50,51,52]. Many studies have shown that antimicrobial resistance would result in higher mortality [53,54]. A plausible explanation for the association difference in mortality between infections with antimicrobial resistant pathogens and non-resistant pathogens may include delay in receiving appropriate antimicrobial therapy. These findings imply that mortality differences across studies are driven by patient vulnerability and pathogen-related factors, highlighting the importance of early stratification of high-risk patients, early detection of antimicrobial-resistant pathogens, and the prudent implementation of antimicrobial stewardship and infection control practices.

This review also highlights important gaps in the literature. Few studies extended beyond phenotypic resistance and examined molecular determinants or genomic profiles of *P. aeruginosa* [24,27,31]. Understanding how virulence genes, resistance mechanisms, and clonal lineages influence clinical outcomes remains an area requiring further investigation. While molecular or genomic studies on PABSI were published before the pandemic, the studies were largely focused on virulence and antibiotic-inactivating enzyme genes [55,56,57]. In contrast, the results of this review indicate that post-pandemic molecular studies of PABSI have begun to expand beyond gene identification, with two studies incorporating sequence type analysis [24,27], illustrating a gradual shift towards more comprehensive genomic epidemiological analyses of PABSI. Our recommendation is that future studies could integrate molecular resistance profiling, including whole-genome sequencing, with standardized clinical data and severity-of-illness scores to determine whether specific resistance mechanisms independently predict mortality in *Pseudomonas aeruginosa* bloodstream infections. Additionally, in only four studies did the authors explicitly collect data extending into the post-pandemic period, and even in these specific cases, the post-COVID-19 data were not analyzed separately [29,31,32,33]. Such factors limit our understanding of how pandemic-related changes in clinical practice may have affected current PABSI patterns.

Lastly, because PABSI is a relatively low-incidence infection, many studies required long data collection periods or multicenter collaborations [14,15,16,17,19,20,21,23,24,25,27,33,34,35], which may explain the slow accumulation of evidence and the limited number of relevant publications within the 2023–2025 window.

This scoping review is constrained by several limitations. Marked heterogeneity among the included studies, such as the definitions of mortality endpoints (e.g., all cause, 7-day, 14-day, 28-day, or 30-day), varied considerably amongst the studies involved. These rates were reported as defined by the original authors and were not standardized across studies, which limited direct comparability. Apart from that, most studies reported risks and outcomes per patient, whereas one reported per episode only [15]. Furthermore, the range of predictors examined differed across the studies and not all were adjusted to the same confounders. As the review was restricted to articles published between 2023 and 2025 to focus on the post-COVID-19 period, the narrow timeframe limited the number of studies to be assessed. Only four studies included data from the post-COVID-19 pandemic era. Although the data were not separated from the results during and before the COVID-19 pandemic, the available evidence suggests that the reported predictors in those studies were largely consistent with the other studies. As a result, only a narrative synthesis could be generated and the relative strength of individual predictors could not be quantified.

Although all studies employed retrospective, observational, cohort designs and most studies used multivariate analyses, many were single-center and outcome definition were heterogenous. Overall study quality is moderate based on these methodological characteristics but as none of the studies were designed specifically to compare pre-, pandemic and post-pandemic periods due to the limited post-pandemic window, a broader comparative analysis would be justified in future.

The geographical distribution of the publications was also imbalanced. Most of the studies originated from Europe and the Western Pacific Region, with particularly strong contributions from Spain, China, and Italy. Regions such as Africa and Southeast Asia were not represented. Overall, the geographical pattern demonstrated a clustering of research in high-income and upper-middle-income regions, with data from lower-resource settings remaining deficient. This imbalance emphasizes the need for greater global surveillance and research efforts to ensure that the findings can be generalized across the diverse healthcare systems in epidemiological contexts. However, as the incidence of this infection is low, a multicenter global approach may be more feasible for data collection.

## 5. Conclusions

In summary, the results of this scoping review demonstrate that severe illness, including sepsis or septic shock, the presence of multidrug or carbapenem-resistant *P. aeruginosa*, mechanical ventilation, carbapenem exposure, high comorbidity burden, hematological disease or malignancy, and corticosteroid therapy remain major predictors of mortality in PABSIs. Carbapenem exposure additionally predisposes patients to acquiring PABSIs, thus reinforcing the importance of antimicrobial stewardship. Despite advances in genomic technologies, molecular predictors for mortality and acquiring disease remain unexplored, highlighting a critical gap to be addressed in future studies. Inclusion of studies from Africa and the Southeast Asia Region would also contribute to closing the aforementioned gap in the current literature. As healthcare systems continue to evolve in the post-COVID-19 pandemic era, further studies integrating clinical, microbiological, and molecular data are needed to refine treatment strategies and improve outcomes for patients with PABSIs. However, as data collection requires extended periods and a multicenter approach to achieve adequate sample sizes, increased international collaboration is required. Systematic reviews based on these different aspects are required in the future to cement the strength of the evidence presented.

## Figures and Tables

**Table 1:** Summary of the studies published from 2023 to 2025.

Study	Country	Setting	Time Period	Number of Patients with Episodes	Mortality Rate	
					30-Day Mortality	7-Day Mortality
[14]	Multinational (Australia, Germany, Greece, France, Israel, Slovenia, Spain, Sweden, and the United Kingdom)	Multicenter (25 centers)	1 January 2009–31 October 2015	464 patients ≥ 80 years studied for risk and mortality Total: 2394 patients	≥80 years (*n* = 464): 30% 65–79 years (*n* = 894): 27% <65 years (*n* = 1036): 25%	-
[15]	Spain	Single center	January 1991–July 2019	2057 episodes	Overall: 20% Solid organ transplant (SOT) patients: 13% Non-SOT: 21%	-
[16]	Japan	Single center	January 2005–December 2020	48 patients	All-cause mortality 32.9%	-
[17]	China	Multicenter (3 centers)	January 2012–December 2020	274 patients	CRPA BSI: 39% DTRPA BSI: 50%	-
[18]	Türkiye	Single center	January 2020–December 2022	140 patients	Overall: 44.2% (*n* = 62) CRPA BSI: 45.5% DTRPA BSI: 45.4%	-
[19]	Türkiye	Multicenter (4 centers)	1 January 2012–31 December 2021	157 patients	44.60%	-
[20]	USA	Single center	1 January 2009–31 December 2022	8039 patients	23.30%	-
[21]	Japan	Single center	January 2009–December 2022	41 patients	75.6% *n* = 31/41	-
[22]	Italy	Single center	January 2020–June 2022	77 patients	20.80%	-
[23]	Multinational (Colombia, Argentina, Italy, Chile, Slovakia, Türkiye, Spain, Brazil, Lebanon, Germany, Switzerland, and the UK)	Multicenter (34 centers)	1 January 2016–31 May 2018	1177 patients	40.30%	27.70%
[24]	Multinational (Spain, Greece, Slovenia, Sweden, and Australia)	Multicenter (6 centers)	2009–2015	836 patients	23.5% (182/773)	15.5% (120/773)
[25]	China	Single center	2013–2022	503 patients	28-day mortality Overall 16.1% CRPA BSI: 38.2% CSPA BSI: 12.2%	-
[26]	Italy	Multicenter (14 centers)	2021 and 2022	285 patients	22.50%	-
[27]	Spain	Multicenter (5 centers)	2006–2018	94 patients	Overall: 32.3% Non-MDR: 37.3% MDR non-XDR: 20% XDR: 17.6%	Overall: 21.5% Non-MDR: 23.9% MDR non-XDR: 10% XDR: 17.6%
[28]	USA	Single center	1 January 2020–30 July 2022	111 patients	*n* = 37/111	-
[29]	China	Single center	January 2017–December 2023	224 patients	CRPA: *n* = 57/112 (50.9%) CSPA: 21.4%	-
[30]	Italy	Multicenter (14 centers)	January 2021–December 2022	511 patients	Overall: *n* = 108/511 (21.1%)	-
[31]	China	Single center	2021–2023	61 patients	44.2% CSPA 50% CRPA	CSPA: 37.2% CRPA: 38.9%
[32]	China	Single center	January 2022–February 2024	118 patients	*n* = 46/118 (38.98%)	-
[33]	Germany	Single center	January 2013–July 2023	50 patients	22% *n* = 11	-
[34]	Spain	Single center	1991–2019	1177 patients	19.8%, *n* = 225	-
[35]	Switzerland	Single center	2015–2021	261 patients and 278 episodes	22% (60 episodes)	14-day mortality: 15% (42 episodes)

**Table 2:** Number of Publications according to Region and Country.

Region and Country	Number of Publications
Americas	
Argentina	1
Brazil	1
Chile	1
Colombia	1
USA	2
Eastern Mediterranean	
Lebanon	1
Europe	
Germany	2
Greece	2
France	1
Israel	1
Italy	4
Slovakia	1
Slovenia	2
Spain	5
Sweden	2
Switzerland	2
Türkiye	3
United Kingdom	2
Western Pacific	
Australia	2
China	5
Japan	2
Southeast Asia	-
Africa	-

**Table 3:** Predictors for mortality due to PABSI based on the studies in the literature from 2023 to 2025.

Risk Factor	Number of Studies
Severity of Illness:	
- Sepsis/septic shock/shock at onset	7
- Mechanical ventilation	5
- ECMO	1
- APACHE II score	3
- Pitt score/qPitt	3
- SOFA score	1
- Multiple organ failure	2
- Inotropic support	1
Microbiological or Antimicrobial Predictors	
- MDR/CRPA	3
- Carbapenem exposure	3
- Use of colistin prior to infection	1
- Cefepime dosing (4 g/day vs. 6 g/day)	1
- PTA > 65%	1
- Incorrect empiric antibiotic therapy	1
- Inadequate empirical antibiotic therapy (EIAT)	1
- High-risk genotypes (ST175, ST235)	1
Host-related Condition and Biomarkers	
- Older age	4
- High comorbidity burden (Charlson Comorbidity Index)	3
- Hematological disease/malignancy	3
- Corticosteroid therapy	3
- Elevated inflammatory markers (PCT/CRP/D-dimer)	2 #
- Thrombocytopenia/low platelet count	2
- Dementia	1
- Acute kidney injury	1
- G-CSF use	1
- Low hemoglobin	1
- Coronary artery disease	1
Infection Source-related Factors	
- Hospital-acquired or -onset	3
- High-risk infection	2
- Urinary tract infection	2 #
- Internal organ infection (pneumonia or cIAI)	1
- Primary bloodstream infection	1
- Lower respiratory tract infection	1
- Persistent bloodstream infection	1
- Urinary catheter	1
Other Risk Factors	
- Source control	2
- Delayed Catheter Removal (>48 h)	1
- COVID-19 pandemic period	1
- Facility complexity (1b vs. 1a)	1

APACHE II, Acute Physiology and Chronic Health Evaluation II; ECMO, Extracorporeal Membrane Oxygenation; cIAI, complicated IntraAbdominal Infection; CRP, C-reactive Protein; CRPA, Carbapenem-Resistant *Pseudomonas aeruginosa*; G-CSF, Granulocyte Colony-Stimulating Factor; MDR, Multidrug-Resistant; PCT, Procalcitonin; SOFA, Sequential Organ Failure Assessment. # All predictors were based on multivariate analysis, except for urinary tract infection by Marco et al. (2025) [34] and elevated inflammatory markers (PCT/CRP/D-dimer) by Çaydaşi et al. (2024) [18].

**Table 4:** Predictors for acquiring PABSIs based on studies in the literature (2023–2025).

Predictors of BSI	Number of Studies
Carbapenem-related exposure (prior therapy/exposure within 90 days)	5
Severe neutropenia (ANC < 100/mm^3^)	2
History of allo-HSCT	1
Older age	1
Receipt of antifungal prophylaxis	1
Active antibiotics during bacteremia	1
Catheter duration > 7 days	1
Differential TTP < 2 h	1
Phlebitis (catheter-related BSI)	1
Positive cultures in both vials	1
Septic shock at BSI onset (catheter-related)	1
Short time-to-positivity (TTP < 13 h)	1
Hematologic malignancy	1
Immunosuppressive therapy	1
Prior ICU hospitalization	1
Chronic lung disease	1
Multiple organ failure	1
Prior CRPA infection/colonization	1
Transplantation	1
Elevated C-reactive protein	1
Central venous catheterization	1

ANC, absolute neutrophil count; BSI, bloodstream infection; CRPA, Carbapenem-Resistant *Pseudomonas aeruginosa*; HSCT, Hematopoietic stem cell transplantation; ICU, intensive care unit; TTP, time-to-positivity.

## Data Availability

The original contributions presented in this study are included in the article/Supplementary Materials. Further inquiries can be directed to the corresponding author.

## Supplementary Materials

The following supporting information can be downloaded at https://www.mdpi.com/article/10.3390/pathogens15010061/s1, Table S1. Preferred Reporting Items for Systematic Reviews and Meta-Analyses extension for Scoping Reviews (PRISMA-ScR) Checklist; Table S2. Searching Strategy for Predictors of Mortality in Pseudomonas aeruginosa Bloodstream Infections between 2023 to 2025.

## Abbreviations

The following abbreviations are used in this manuscript:

PABSI	*Pseudomonas aeruginosa* bloodstream infection
ICU	intensive care unit
WHO	World Health Organization
CRPA	carbapenem-resistant *P. aeruginosa*
CSPA	carbapenem-susceptible *P. aeruginosa*
PRISMA-ScR	Preferred Reporting Items for Systematic Reviews and Meta-Analyses Extension for Scoping Reviews
ECMO	extracorporeal membrane oxygenation
APACHE	Acute Physiology and Chronic Health Evaluation
SOFA	Sequential Organ Failure Assessment
MDR	multi-drug resistant
PTA	probability of target attainment
PCT	procalcitonin
CRP	C-reactive protein
ANC	absolute neutrophil count
TTP	time-to-positivity
DTRPA	Difficult-to-treat *P. aeruginosa*
