# Supplementary material for: Predictors of Mortality in Pseudomonas aeruginosa Bloodstream Infections: A Scoping Review
Source: Pathogens. 2026 Jan 7;15(1):61. doi: 10.3390/pathogens15010061 (PMC12844950; doi:10.3390/pathogens15010061)
Supplement: Supplementary file 1 [file pathogens-15-00061-s001.zip › Table S2. Searching Strategy for Predictors of Mortality in Pseudomonas aeruginosa Bloodstream Infections between 2023 to 2025.pdf]

Supplementary Table S2. Searching Strategy for Predictors of Mortality in Pseudomonas aeruginosa Bloodstream Infections between 2023 to 2025

**PubMed**
("Risk Factor"[Mesh] OR "Risk Factors"[Mesh] OR "Mortality"[Mesh]) AND ("Pseudomonas aeruginosa"[Mesh]) AND ("Bacteremia"[Mesh] OR "Bloodstream Infections"[Mesh]) Filters: from 2023-2025
**Scopus**
((TITLE-ABS-KEY (Risk Factor) OR TITLE-ABS-KEY (Risk Factors)) AND TITLE-ABS-KEY (Mortality) AND (TITLE-ABS-KEY ("Pseudomonas aeruginosa") OR TITLE-ABS-KEY ("P. aeruginosa")) AND (TITLE-ABS-KEY ("Bloodstream infection") OR TITLE-ABS-KEY ("Bloodstream infections") OR TITLE-ABS-KEY ("Blood stream infection") OR TITLE-ABS-KEY (bacteremia))) AND PUBYEAR > 2023 AND PUBYEAR < 2026
**Web of Science**
TS=(("Risk Factor" OR "Risk Factors")<br>AND<br>("Mortality")<br>AND<br>("Pseudomonas aeruginosa" OR "P. aeruginosa")<br>AND<br>("Bloodstream infection" OR "Bloodstream infections"<br>OR "Blood stream infection" OR bacteremia))<br>AND PY=(2023-2025)
**Embase**
'Pseudomonas aeruginosa' AND ('risk factor' OR 'risk factors') AND 'mortality' AND ('bloodstream infection' OR 'bloodstream infections' OR 'bacteremia') AND (2023:py OR 2024:py OR 2025:py)
