# Supplementary material for: Predictors of Mortality in Pseudomonas aeruginosa Bloodstream Infections: A Scoping Review
Source: Pathogens. 2026 Jan 7;15(1):61. doi: 10.3390/pathogens15010061 (PMC12844950; doi:10.3390/pathogens15010061)
Supplement: Supplementary file 1 [file pathogens-15-00061-s001.zip › Table S1.PRISMA-ScR for PABSI.pdf]

Supplementary Table S1. Preferred Reporting Items for Systematic Reviews and Meta-Analyses extension for Scoping Reviews (PRISMA-ScR) Checklist

SECTION	ITEM	PRISMA-ScR CHECKLIST ITEM	REPORTED ON PAGE #
TITLE	1	Predictors of mortality and acquiring Pseudomonas aeruginosa bloodstream infections: A scoping review of publications post-COVID19 pandemic	Page 1
ABSTRACT			
Structured summary	2	Background: Pseudomonas aeruginosa bloodstream infections	Page 1
INTRODUCTION			
Rationale	3	The COVID-19 pandemic altered the patterns of antimicrobial use which led to downstream effects of bacterial infections. Numerous studies have been published during the pandemic but very few distinguished pre-pandemic, pandemic and post-pandemic data. This scoping review fills in the knowledge gap and lays the groundwork for future comparative analyses.	Page 2
Objectives	4	The review aims to (1) identify recent predictors of mortality in PABSI, (2) describe risk factors associated with acquiring PABSI, (3) summarise recent mortality rates across diverse clinical settings, and (4)	Page 2

		characterise the geographical distribution of published research.	
METHODS			
Protocol and registration	5	No review protocol was registered for this scoping review	“Scoping review was not registered”
Eligibility criteria	6	Publications from 2023-2025, in English, peer-reviewed	Page 3
Information sources	7	PubMed, Scopus, Web of Science, and EMBASE	Page 3
Search	8	Mesh Terms have been listed in Supplementary Table 2	Page 3
Selection of sources of evidence	9	Screening of titles, abstracts and publication years was performed according to predefined criteria; duplicates were removed, title/abstract and full-text were reviewed using predefined inclusion and exclusion criteria. Full-text were reviewed independently by two authors and disagreements were resolved amicably by consensus or by a third author.	Page 3
Data charting process	10	Data was charted independently by two reviewers using an Excel sheet formed by the authors and any discrepancies were resolved during discussion.	Page 3
Data items	11	Reference (First author and study title), study site, period of study, clinical setting, number of patients,	Page 3

		predictors of mortality and acquiring PABSI, and mortality rate.	
Critical appraisal of individual sources of evidence	12	Critical appraisal was not done as this is a scoping review	
Synthesis of results	13	Data was summarized using narrative synthesis and tabulation of the studies based on the geographical distribution of studies, predictors of mortality, mortality rates and predictors of acquiring PABSI.	Page 3
RESULTS			
Selection of sources of evidence	14	A total of 869 articles were screened; 22 met eligibility criteria and were included. Exclusions were due to duplication or did not meet criteria	Page 4
Characteristics of sources of evidence	15	Data was charted on year of publication. Extracted variables included study site, period of study, clinical setting, population size, predictors of mortality, predictors of acquiring PABSI, and mortality rates. Full citations are included in reference list.	Page 5 and 6
Critical appraisal within sources of evidence	16	Critical appraisal was not done as this is a scoping review	
Results of individual sources of evidence	17	Data on the predictors of mortality and acquiring PABSI, common risk factors and region-specific profiles of the locations of the studies were tabulated in separate	Page 5 and 6

		tables to address the objectives.	
Synthesis of results	18	Tabulated data summarized the risk factors for mortality and acquiring PABSI to address the objectives	Table 3 and Table 4
DISCUSSION			
Summary of evidence	19	Summary of main results and linking key results with known relevant evidence.	Page 13 and 14
Limitations	20	Marked heterogeneity of studies prevented quantification of predictors. Only four studies have included the time period during post-COVID19 pandemic but the data was not analyzed separately.	Page 15
Conclusions	21	Sepsis or septic shock, presence of multidrug or carbapenem-resistant *P. aeruginosa*, mechanical ventilation, carbapenem exposure, high comorbidity burden, hematological disease or malignancy and corticosteroid therapy remain major predictors of mortality in PABSI, whereas carbapenem exposure is a predictor for PABSI acquisition.	Page 15
Funding			
Funding	22	No external funding was received for this review	Page 16
